# Changes in antibiotic prescribing by dentists in the United States, 2012–2019

**DOI:** 10.1017/ice.2023.151

**Published:** 2023-11

**Authors:** Swetha Ramanathan, Connie H. Yan, Colin Hubbard, Gregory S. Calip, Lisa K. Sharp, Charlesnika T. Evans, Susan Rowan, Jessina C. McGregor, Alan E. Gross, Ronald C. Hershow, Katie J. Suda

**Affiliations:** 1 School of Public Health, University of Illinois at Chicago, Chicago, Illinois; 2 College of Pharmacy, University of Illinois at Chicago, Chicago, Illinois; 3 Department of Medicine, University of California San Francisco, San Francisco, California; 4 Center of Innovation for Complex Chronic Healthcare, Hines Veterans’ Affairs Hospital, Hines, Illinois; 5 Center for Health Services and Outcomes Research, Northwestern University Feinberg School of Medicine, Chicago, Illinois; 6 College of Dentistry, University of Illinois at Chicago, Chicago, Illinois; 7 College of Pharmacy, Oregon State University, Portland, Oregon; 8 Department of Medicine, University of Pittsburgh, Pittsburgh, Pennsylvania; 9 Center for Health Equity Research and Promotion, Veterans’ Affairs Pittsburgh Healthcare System, Pittsburgh, Pennsylvania

## Abstract

**Objectives:**

Dentists prescribe 10% of all outpatient antibiotics in the United States and are the top specialty prescriber. Data on current antibiotic prescribing trends are scarce. Therefore, we evaluated trends in antibiotic prescribing rates by dentists, and we further assessed whether these trends differed by agent, specialty, and by patient characteristics.

**Design:**

Retrospective study of dental antibiotic prescribing included data from the IQVIA Longitudinal Prescription Data set from January 1, 2012 to December 31, 2019.

**Methods:**

The change in the dentist prescribing rate and mean days’ supply were evaluated using linear regression models.

**Results:**

Dentists wrote >216 million antibiotic prescriptions between 2012 and 2019. The annual dental antibiotic prescribing rate remained steady over time (*P* = .5915). However, the dental prescribing rate (antibiotic prescriptions per 1,000 dentists) increased in the Northeast (by 1,313 antibiotics per 1,000 dentists per year), among oral and maxillofacial surgeons (n = 13,054), prosthodontists (n = 2,381), endodontists (n = 2,255), periodontists (n = 1,961), and for amoxicillin (n = 2,562; *P* < .04 for all). The mean days’ supply significantly decreased over the study period by 0.023 days per 1,000 dentists per year (*P* < .001).

**Conclusions:**

From 2012 to 2019, dental prescribing rates for antibiotics remained unchanged, despite decreases in antibiotic prescribing nationally and changes in guidelines during the study period. However, mean days’ supply decreased over time. Dental specialties, such as oral and maxillofacial surgeons, had the highest prescribing rate with increases over time. Antibiotic stewardship efforts to improve unnecessary prescribing by dentists and targeting dental specialists may decrease overall antibiotic prescribing rates by dentists.

In the United States, 10% of outpatient antibiotic prescriptions are written by dentists.^1^ Studies on dental antibiotic prescribing have found that penicillins are the most commonly prescribed antibiotic by dentists; they account for ∼70% of all dental antibiotic prescriptions.^2^ Dentists also prescribe other classes of antibiotics not commonly prescribed by other providers, such as clindamycin.^2,3^ Infection prophylaxis is the most common reason for antibiotics prescribed by dentists, 77.6% of which are discordant with guidelines in the United States.^4^ Dentists also prescribe antibiotics for the treatment of acute oral infections, among which, 12%–28% of prescriptions are unnecessary.^5^

Guidelines provide recommendations for antibiotic use in dental practice to minimize unnecessary antibiotic prescribing. Antibiotic-specific guidelines include the 2021 American Heart Association (AHA) guidelines^6,7^ and the 2013 American Academy of Orthopedic Surgeons guidelines^8^ on infection prophylaxis for patients with specific cardiac diagnoses and prosthetic joints, respectively, as well as the 2019 guidelines for management of pulpal- and periapical-related dental pain and intraoral swelling. Targeted antibiotic stewardship in dental settings have also been implemented to assist with improving guideline-concordant antibiotic use.^9^

A recent study of US outpatient antibiotic prescribing between 2011 and 2016 suggests increasing trends in dental antibiotic prescribing, whereas prescribing by medical clinicians has decreased.^10^ However, data from 2015–2017 from the Department of Veterans’ Affairs suggest that dental antibiotic prescribing is decreasing.^11^ The primary objective of this study was to evaluate trends in antibiotic prescribing rates by dentists between 2012 and 2019. A secondary objective was to assess whether these trends differed by antibiotic agent and dentist specialty.

## Methods

### Study design and data source

In this cross-sectional retrospective study of dental antibiotic prescribing, we evaluated data from the IQVIA Longitudinal Prescription Data set (LRx) from January 1, 2012, to December 31, 2019. LRx includes 92% of all dispensed outpatient prescriptions in the United States. LRx contains prescriptions dispensed without insurance reimbursement and those reimbursed by public and private payers. This analysis was performed at the prescription level and included all dispensed prescriptions prescribed by all dentists as well as hygienists and technologists. This data set included prescription data (date dispensed, generic drug name, national drug code [NDC], strength, dosage form, and days’ supply), prescriber data (prescriber identifier, type, specialty, 5-digit ZIP code, and state of the practice location), and patient data (age and sex). Antibiotics were limited to oral forms (eg, capsules or tablets) or liquid systemic forms (liquid dosage forms). If a prescription was missing information regarding any prescription, patient, or prescriber information, the prescription was excluded. This research was reviewed and approved by the university’s institutional review board.

### Outcomes

Dental antibiotic prescribing trends were evaluated using total number of antibiotics prescribed, mean days’ supply, and an annual provider-based prescribing rate. The annual provider-based prescribing rate was calculated by dividing the number of antibiotics prescribed each year by the number of actively prescribing dentists per year (reported as the “provider-based prescribing rate” herein). This number was reported as the prescribing rate per 1,000 dentists per year and the percentage change per year. Actively prescribing dentists were defined as those who prescribed ≥20 of any prescription in a calendar year within the data set.^1^ Mean days’ supply was calculated for each year for the number of prescriptions that were prescribed.

### Covariates

Additional variables to describe the cohort included patient age (1–17 years, 18–39 years, 40–64 years, or ≥65 years), patient sex (male or female), and US Census Bureau Region (Northeast, South, Midwest, or West). Antibiotics were classified as amoxicillin, clindamycin, cephalexin, azithromycin, penicillin, doxycycline, fluoroquinolones, and other (eg, erythromycin, clarithromycin, linezolid, metronidazole, nitrofurantoin, trimethoprim, etc). Amoxicillin-clavulanic acid was combined with the amoxicillin category. Dentists were categorized as general dentists, anesthesiologists, endodontists, orthodontists, periodontists, prosthodontists, oral and maxillofacial surgeons, pediatric dentists, and hygienists or technologists according to dental specialty data within the LRx data set.

### Statistical analysis

The frequency distribution of prescription data was examined according to patient, provider, and prescription variables. Means with standard deviations were calculated for the provider-based prescribing rate and days’ supply. Linear regression models were used to analyze the annual change in the provider-based prescribing rate, total antibiotics prescribed, and mean days’ supply. We used linear regression models to assess trends across all antibiotics, by adult (≥18) and children’s (<18) prescriptions, by antibiotic agent, geography, and dental specialties. Statistical significance was defined as *P* < .05. Because large data sets may identify small differences to be statistically significant, we defined clinically important results as differences ≥5%. All analyses were conducted using SAS version 9.4 software (SAS Institute, Cary, NC).

## Results

After removing prescriptions for missing information (N = 3,946,827, 1.7% of total), >216 million antibiotics were prescribed by 241,106 dentists from January 2012 to December 2019. Most prescriptions were for male patients (55.0%) and for those aged 40–64 years (42.0%). Most prescribing dentists were located in the Southern region (38.7%) and were categorized as general dentists (86.7%) or oral and maxillofacial surgeons (12.2%) (Table 1). Other dental specialists prescribed <1% of all antibiotics. Amoxicillin and clindamycin were the most prevalent antibiotics prescribed (62.8% and 14.2%, respectively) (Table 2).

**Table 1.** Provider-Based Prescribing Rate by Dental Specialty, Geographic Region, and Payer Type

Variable	Total Prescriptions, No. (%)	Provider-Based Prescribing Rate (SD)^a^	Annual Change in the Provider-Based Prescribing Rate (95% CI)^b^	*P* Value	% Change in Prescribing Rate Per Year
Overall		142,155.4 (3,421.2)	314.8 (−1,044.5 to 1674.1)	.5915	0.2
**Geographic region**					
Northeast	40,818,739 (18.8)	132,779.90 (3,951.96)	1,313.66 (378.01 to 2,249.314)	.0139	1.0
Midwest	44,807,801 (20.7)	147,192.31 (4,163.26)	−56.45 (−1,753.37 to –1,640.46)	.9378	–0.04
South	83,876,370 (38.7)	167,667.86 (3,794.52)	−603.39 (−2,028.65 to 821.87)	.3402	–0.4
West	47,012,012 (21.7)	114,430.32 (3,841.07)	730.54 (−655.54 to 2,116.62)	.2446	0.6
**Dental specialty**					
General	187,756,760 (86.7)	129,466.02 (3,052.97)	105.866 (−1,134.69 to 1,346.42)	.8415	0.08
Dental anesthesiologist	3,158 (<0.01)	20,904.27 (16,521.82)	1410.71 (−5,178.19 to 7,999.60)	.6191	6.7
Endodontist	902,057 (0.42)	184,291.35 (71,96.97)	2255.91 (375.45 to 4,136.37)	.0261	1.2
Orthodontist	37,974 (0.02)	12,848.92 (2,860.80)	−1,062.38 (−1,547.02 to −577.75)	<.001	−8.3
Periodontist	1,024,212 (0.4)	254,556.85 (6,234.72)	1,961.46 (341.04 to 3,581.88)	.0252	0.8
Prosthodontist	45,099 (0.02)	84,123.15 (7,127.93)	2381.75 (711.63 to 4,051.88)	.0130	2.8
Oral and maxillofacial surgery	26,462,590 (12.2)	497,190.97 (33,169.39)	13,054 (9,456.09 to 16,651)	<.001	2.6
Pediatric	280,978 (0.1)	42,854.77 (1,755.82)	−299.28 (−949.94 to 351.37)	.3034	−0.7
Hygienist/Technologist	2,094 (<0.01)	5,783.60 (2,951.98)	255.15 (−921.44 to 1,431.73)	.6147	4.4

Note. CI, confidence interval; SD, standard deviation.

a Average annual prescribing rate per 1,000 dentists per year.

b The annual change in the provider-based prescribing rate per 1,000 dentists per year. Positive numbers indicate a mean annual increase in the prescriber-based prescribing rate over the study period. Negative numbers indicate a mean annual decrease in the prescriber-based prescribing rate over the study period.

**Table 2.** Changes in Provider-Based Antibiotic Prescribing Rates, Total Antibiotics Prescribed per Year, and Mean Days’ Supply Overall and by Adults and Antibiotic Agent

Variable	Prescriptions (%)	Annual Provider-Based Prescribing Rate (SD)^a^	Annual Change in the Provider-Based Prescribing Rate (95% CI)^a^	*P* Value	Percent Change in Prescribing Rate per Year	Total Antibiotics Prescribed per Year (95% CI)^a^	*P* Value	Mean Days’ Supply (SD)^b^	*P* Value
Overall	216,514,922	142,155.4 (3,421.2)	314.8 (−1,044.5 to 1,674.1)	.5915	0.2	242,277 (−36,026 to 520,579)	.0772	7.3 (0.06)^c^	<.001
Adults	202,568,109 (93.6)	135,261.7 (3,542.8)	560.0 (−771.9 to 1,891.9)	.3433	0.4	259,982 (−8,168.58.0 to 528,133)	.0553	7.2 (0.06)^c^	<.001
Children		13,708.3 (178.9)	−49.3 (−103.1 to 4.6)	.0665	−0.4	−17,706 (−28,934 to −6,477.6)	.0084	7.83 (0.02)^c^	.0170
**Antibiotic agent**									
Amoxicillin	136,013,952 (62.8)	92,657.3 (6,638.2)	2,562.7 (1,682.1 to 3,443.2)	<.001	2.7	609,245 (447,382 to 771,108)	<.001	7.2 (0.03)	.2165
Clindamycin	30,744,005 (14.2)	24,130.4 (860.1)	−41.0 (−389.4 to 307.4)	.7829	−0.2	21,300 (−39,983 to 82,583)	.4277	7.0 (0.02)	.4899
Cephalexin	8,642,004 (4.0)	12,186.9 (855.3)	−328.0 (−447.7 to −208.2)	<.001	−2.7	−494,073 (−624,028 to −364,119)	<.001	7.2 (0.1)^c^	<.001
Azithromycin	9,034,909 (4.2)	9,580.4 (530.6)	−151.7 (−306.2 to 2.8)	.0532	−1.6	−22,845 (−48,716 to 3,025.4)	.0740	5.0 (0.004)	.3322
Penicillin	24,366,053 (11.3)	7,651.8 (754.6)	−894.5 (−1,180.6 to −608.4)	<.001	−11.7	−25,639 (−30,819 to −20,459)	<.001	7.4 (0.02)^c^	.0119
Doxycycline	2,558,179 (1.2)	3,487.9 (259.5)	−305.4 (−346.3 to −264.4)	<.001	−8.7	−13,398 (−185,71 to −8,225.9)	<.001	18.4 (0.5)^c^	.0378
Fluroquinolones	1,355,599 (0.6)	34,651.7 (2,300.6)	−91.4 (−144.9 to −37.9)	.0058	−0.3	−240,635 (−266,999 to −214,272)	<.001	8.7 (0.08)^c^	<.001
Other	3,800,221 (1.8)	5,590.8 (3,82.3)	−150.5 (−191.9 to −109.1)	<.001	−2.7	−36,677 (−39,457 to −33,897)	<.001	8.6 (0.08)	.4311

Note. CI, confidence interval; SD, standard deviation.

a Rates are per 1,000 dentists per year.

b Significantly decreasing over time.

c Prescribing rate is per 1,000 dentists per year.

### Overall trends

The average provider-based prescribing rate between 2012 and 2019 was 142,155 prescriptions per 1,000 dentists per year (Table 1). There was no significant trend in the provider-based prescribing rate for all antibiotics between 2012 and 2019 (*P* = .5915) (Table 1). However, the mean days’ supply significantly decreased over the study period by 0.023 days per 1,000 dentists per year (95% CI, −0.017 to −0.030; *P* < .001) or 2% per year over the 7-year period. Furthermore, the annual number of prescriptions increased by 10.4% from 2012 to 2017 (Supplementary Table S2 online). However, between 2017 and 2019, the number of prescriptions decreased by 3.2% (Supplementary Table S2).

### Trends by geographic region and dental specialty

**Figure 1.**
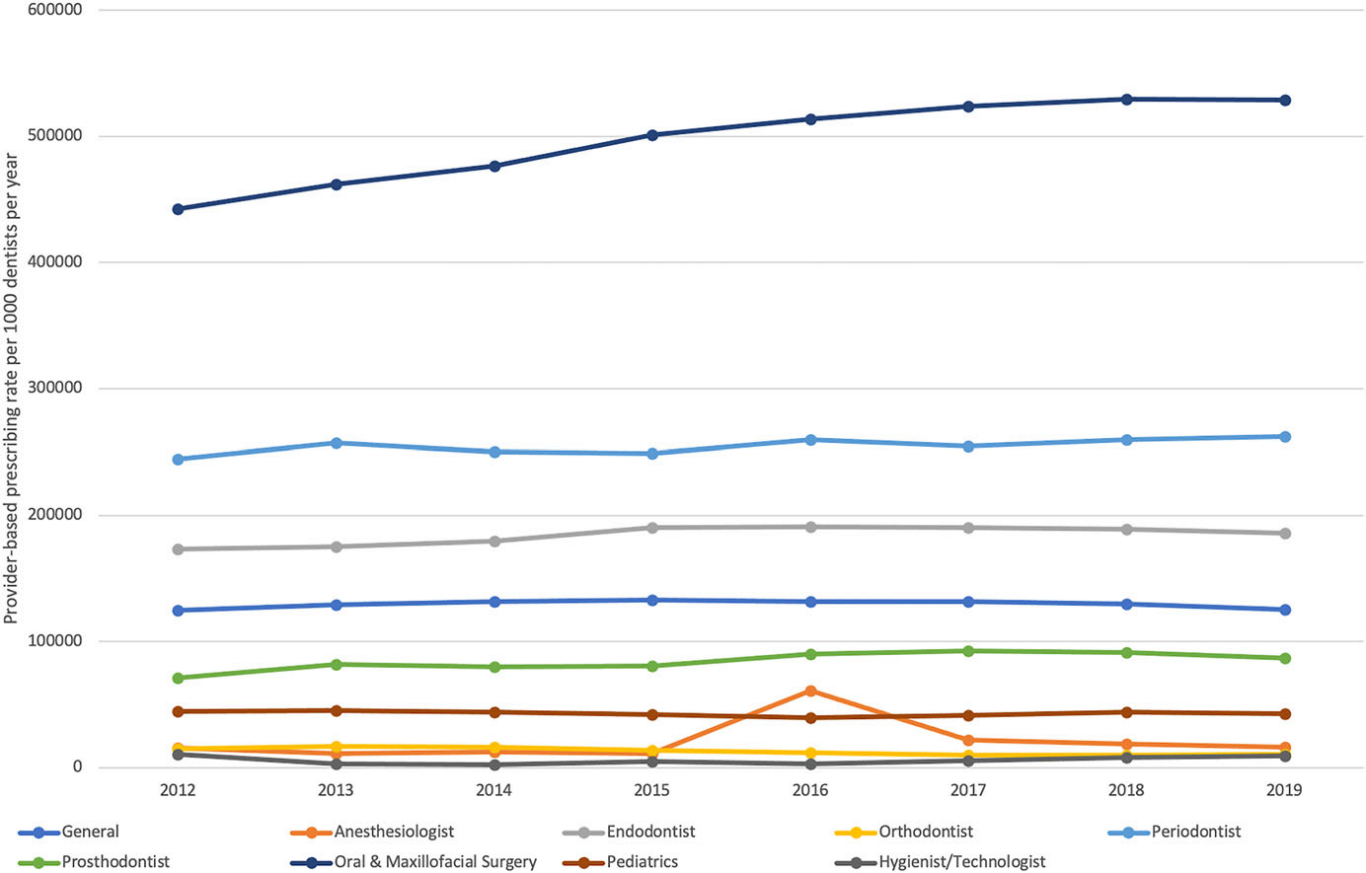
Changes in yearly dentist prescribing rate by dental specialties from 2012 to 2019. ^a^Prescribing rate is per 1,000 dentists per year. ^b^Includes oral tablets or capsules and liquid antibiotics in both adults and children. ^c^Prescribing rates increased significantly for endodontists, periodontists, prosthodontists, and oral and maxillofacial surgeons, but decreased significantly for orthodontists.

Trends in provider-based prescribing varied by geographic region and dental specialty. The highest provider-based prescribing rate was in the South, followed by the Midwest, Northeast, and West (Table 1). Prescribing rates significantly increased in the Northeast, but rates in the Southern, Midwestern, and Western regions did not change over time (Table 1). Dental specialists, especially oral and maxillofacial surgeons, periodontists, dental anesthesiologists, and endodontists had the highest provider-based prescribing rates (Table 1). Linear regression analyses indicated that the provider-based prescribing rate significantly increased for endodontists, periodontists, prosthodontists, and oral and maxillofacial surgeons. The provider-based prescribing rate decreased for orthodontists. Interestingly, the provider-based prescribing rate for dental anesthesiologists temporarily increased in 2016 (Fig. 1).

### Trends among adults and children

During the study period, ∼202 million (93.6%) of the antibiotics prescribed by dentists were prescribed for adults and 13 million (6.4%) were prescribed for children (Table 2 and Supplementary Table S3 online). Similar to overall trends, the most commonly prescribed antibiotics for adults and children were amoxicillin (62.1% and 73.2%, respectively), clindamycin (14.6% and 7.7%), and penicillin (1.2% and 12.0%) (Supplementary Table S3).

Among adults, the provider-based prescribing rate was 135,261 (SD, 3,542) per 1,000 dentists per year at a rate of 0.4% per year and did not significantly increase over the 7-year period (*P* = .3433) (Table 2). Among adults, the mean days’ supply (7.2 days; SD, 0.06) significantly decreased by 0.024 days per 1,000 dentists per year (95% CI, −0.017 to −0.030; *P ≤* .001) for adult prescriptions between 2012 and 2019 (Table 2).

Among children, the mean provider-based prescribing rate for all years was 13,708 (SD, 178.9) per 1,000 dentists per year, and with a nonsignificant decrease (−49.3 prescriptions per 1,000 dentists per year; *P* = .0665) over the study period (Supplementary Table S1 online). The number of antibiotics prescribed significantly decreased at a yearly rate of 17,706 antibiotics per 1,000 dentists per year among children (*P* = .0084), which was equivalent to a −0.4% change (decrease) in prescribing rate per year. The mean days’ supply for antibiotics prescribed to children (7.8 days; SD, 0.02) decreased significantly by 0.0077 days per 1,000 dentists per year (95% CI, −0.0019 to −0.013; *P* = .0170) between 2012 and 2019.

### Trends by antibiotic agent

Amoxicillin was consistently the most commonly prescribed antibiotic across each of the 8 years (Supplementary Table S2 online). Although penicillin was the second most prescribed antibiotic in 2012, from 2013 through 2019 clindamycin was the second most prescribed antibiotic (Supplementary Table S2). The annual provider-based prescribing rate of amoxicillin increased by 2,562.7 prescriptions per 1,000 dentists per year (*P ≤* .001), whereas it decreased for cephalexin, penicillin, doxycycline, fluoroquinolones, and other antibiotics at rates of −328, −894.5, −305.4, −91.4, and −150.50 prescriptions per 1,000 dentists annually, respectively (Table 2). No significant trends were observed for clindamycin and azithromycin. Data regarding trends over time for days’ supply are listed in Table 2. Azithromycin had the shortest mean days’ supply (5.0 days) and doxycycline had the longest (18.4 days). The total number of antibiotics prescribed per year decreased for cephalexin, penicillin, fluoroquinolones, and other antibiotics. However, the total number of amoxicillin prescriptions prescribed increased by 609,247 prescriptions per 1,000 dentists per year, equivalent to a rate of 2.7% per year (Table 2).

## Discussion

Over the study period, the antibiotic prescribing rate per 1,000 dentists remained steady, whereas the mean days’ supply for all antibiotics decreased. This finding suggests that although the prescribing rate was unchanged over the study period, the days’ supply decreased and, thus, fewer doses of antibiotics prescribed by dentists circulated in the community. Considering that the majority of antibiotics prescribed by dentists are for preventative reasons,^12^ fewer patients may have a supply of antibiotics at home (ie, for future dental appointment, self-prescription). This finding also suggests dentist concordance with recent guidelines recommending shorter durations.^13^

Previous research has described trends in antibiotic prescribing by all provider types, including dentists. We previously identified a decrease in dental antibiotic prescribing between 2005 and 2010, from 10.7% of all antibiotics prescribed in the community in 2005 to 10% in 2010.^2^ However, a more recent national analysis by King et al^10^ found that dental prescribing increased while medical clinician prescribing decreased between 2011 and 2016. In our study with a longer timeframe of data through 2019, trends in the dental antibiotic prescribing rate remained steady and were discordant with national decreases observed for medical clinicians.^14^

Similar to results in our population-level cohort, Durkin et al^15^ identified the highest proportion of prescriptions among general dentists followed by oral and maxillofacial surgeons in a commercially insured cohort. Importantly, oral and maxillofacial surgeons had the highest prescribing rate in our study. Although oral and maxillofacial surgeons prescribed 12% of all antibiotics over the study period, these dental specialists only account for 4% of all dentists in the United States.^16^ With the exception of orthodontists, large increases in the prescribing rate were observed for dental specialists, especially oral and maxillofacial surgeons, endodontists, periodontists, and prosthodontists. We also identified a temporary increase in antibiotic prescribing by dental anesthesiologists in 2016. The reason for the increases in prescribing rate by dental specialists is unclear, and a lack of visit-level data made it difficult to decipher the cause.^17^ Potential explanations for increased prescribing by dental specialists include the increased use of dental implants and worsening oral health in an aging population.^18^ To our knowledge, this is the first description of antibiotic prescribing trends by dental specialists.

This study had several limitations. The IQVIA LRx data set did not include characteristics of dental visits or patient comorbidities. Thus, we could not evaluate the appropriateness of the antibiotic prescribed. Furthermore, we included all prescriptions dispensed, but we could not determine whether patients actually took the antibiotic. Finally, the data included in this study may not include all patients receiving dental care because the LRx database only contains information on dispensed prescriptions.

Regardless, our results are relevant to public health efforts to preserve the effectiveness of antibiotics. Obtaining data identifying the indication for a dental antibiotic is challenging.^19^ Population-level prescription data sets, such as IQVIA LRx, can be used to monitor the need for and effectiveness of antibiotic stewardship strategies.^20^ The CDC Core Elements of Outpatient Antibiotic Stewardship recommend the use of prescribing data to inform stewardship efforts.^20^ Although dentists are included as a targeted group to implement the Core Elements, there are few examples of the implementation of antibiotic stewardship in dental practices. These antibiotic stewardship strategies should be tailored to the practice of dental specialists, especially oral and maxillofacial surgeons. Future monitoring should assess overall antibiotic prescribing rates, trends by dental specialists, and the impact of removing clindamycin from the 2021 update to the AHA infective endocarditis prophylaxis guidelines.^7^ Before-and-after analyses specific to guideline changes and/or stewardship efforts that are implemented as a result of these data can also be monitored with the use of IQVIA LRx to determine whether these strategies have made a significant impact. Using these data in this manner can be an effective monitoring strategy in future dental stewardship efforts. Antibiotics are commonly used as premedication to prevent complications from tooth extractions and dental implants.^12^ Although data are mixed on the effectiveness of this practice,^21^ guidelines recommending antibiotic prophylaxis for extractions and implants are not available. The United Kingdom does not recommend antibiotics to prevent postoperative infection or dry socket associated with tooth extraction and only recommends antibiotics to prevent dental implant complications when bone augmentation is part of the procedure.^22^ Finally, the impact of the 2015 ADA guidelines removing antibiotic prophylaxis recommendations in patients with prosthetic joints appears to have minimal, if any, impact on overall antibiotic prescribing rates by dentists.^23^

In conclusion, between 2012 and 2019, provider-based prescribing rates for all antibiotics prescribed by dentists remained steady, which is discordant with medical clinician prescribing trends. However, mean days’ supply for antibiotics prescribed by dentists did decrease over time. Oral and maxillofacial surgeons had the highest prescribing rate, with increases over time. Antibiotic stewardship efforts to improve unnecessary prescribing by dentists and targeting dental specialists and prescribing of clindamycin may decrease overall antibiotic prescribing rates by dentists.

## Supplementary material

For supplementary material accompanying this paper visit https://doi.org/10.1017/ice.2023.151. click here to view supplementary material
