# Supplementary material for: Changes in antibiotic prescribing by dentists in the United States, 2012–2019
Source: Infect Control Hosp Epidemiol. 2023 Aug 22;44(11):1725–30. doi: 10.1017/ice.2023.151 (PMC10665869; doi:10.1017/ice.2023.151)
Supplement: Supplementary file 1 [file S0899823X23001514sup001.docx]

Table S1. Changes in provider-based antibiotic prescribing rates, total antibiotics prescribed per year, and mean days’ supply overall by children.

|  | Prescriptions (%) | Annual provider-based prescribing rate per 1000 dentists per year (SD) | Annual change in the provider-based prescribing rate^a^ (95% CI) | p-value | Percent change in prescribing rate per year | Total antibiotics prescribed per year | p-value | Mean days’ supply (SD)^b^ | p-value |
| --- | --- | --- | --- | --- | --- | --- | --- | --- | --- |
| Children | 13,946,813 (6.4) | 13,708.3 (178.9) | -49.3 (-103.1, 4.6) | 0.0665 | -0.4% | -17,706 (-28,934, -6,477.6) | 0.0084 | 7.83 (0.02) ^†^ | 0.0170 |

Table S2. Annual trends in antibiotics dispensed from outpatient pharmacies prescribed by dentists, 2012-2019.

|  | 2012 | 2013 | 2014 | 2015 | 2016 | 2017 | 2018 | 2019 |
| --- | --- | --- | --- | --- | --- | --- | --- | --- |
| Total^a^ | 25,197,085 (11.6) | 26,355,163 (12.2) | 27,197,360 (12.6) | 27,723,785 (12.8) | 27,641,470 (12.8) | 27,829,662 (12.9) | 27,650,361 (12.8) | 26,920,036 (12.4) |
| Antibiotic Agent |  |  |  |  |  |  |  |  |
| Amoxicillin | 14,261,724 (56.6) | 15,424,086 (58.5) | 16408113 (60.3) | 17159246 (61.9) | 17,548,016 (63.5) | 18,125,272 (65.1) | 18,511,821 (67.0) | 18,575,674 (69.0) |
| Clindamycin | 3,553,392 (14.1) | 3,760,111 (14.3) | 3908270 (14.4) | 3995581 (14.4) | 3,975,981 (14.4) | 3,961,365 (14.2) | 3,900,646 (14.1) | 3,688,659 (13.7) |
| Cephalexin | 1,191,956 (4.7) | 1,218,502 (4.6) | 1190415 (4.4) | 1133759 (4.1) | 1,060,448 (3.8) | 1,017,847 (3.7) | 949,247 (3.4) | 879,830 (3.3) |
| Azithromycin | 1,187,007 (4.7) | 1,140,908 (4.3) | 1148678 (4.2) | 1177824 (4.3) | 1,176,979 (4.3) | 1,168,822 (4.2) | 1,103,926 (4.0) | 930,765 (3.5) |
| Penicillin | 3,781,692 (15.0) | 3,646,669 (13.8) | 3458650 (12.7) | 3250182 (11.7) | 2,968,002 (10.7) | 2,712,075 (9.8) | 2,413,879 (8.7) | 2,134,904 (7.9) |
| Doxycycline | 430,191 (1.7) | 384,526 (1.5) | 346306 (1.3) | 320793 (1.2) | 294,970 (1.1) | 276,347 (1.0) | 259,667 (0.9) | 245,379 (0.9) |
| Fluoroquinolone | 193,849 (0.8) | 205,814 (0.8) | 200890 (0.7) | 191725 (0.7) | 173,009 (0.6) | 147,799 (0.5) | 129,519 (0.5) | 112,994 (0.4) |
| Other | 597,274 (2.4) | 574,547 (2.2) | 536038 (1.9) | 494675 (1.8) | 444,065 (1.6) | 420,135 (1.5) | 381,656 (1.4) | 351,831 (1.3) |

^a^ Percentages reflect the proportion of antibiotics dispensed by dentists per year.

Table S3. Antibiotics prescribed to Adults and Children by dentists, 2012-2019 (total=216,514,922)

| Antibiotic Class | Adults | Children |
| --- | --- | --- |
|  | Frequency (%) | Frequency (%) |
| Total | 202,568,109 | 13,946,813 |
| Amoxicillin | 125,802,879 (62.1) | 10,211,073 (73.2) |
| Clindamycin | 29,675,567 (14.6) | 1,068,438 (7.7) |
| Cephalexin | 8,295,583 (4.1) | 346,421 (2.5) |
| Azithromycin | 8,620,470 (4.3) | 414,439 (3.0) |
| Penicillin | 22,696,192 (1.2) | 1,669,861 (12.0) |
| Doxycycline | 2,515,456 (1.2) | 42,723 (0.3) |
| Fluoroquinolone | 1,336,713 (0.7) | 18,886 (0.1) |
| Other | 3,625,249 (1.8) | 174,972 (1.3) |
